# Disappointing success of electrical cardioversion for new-onset atrial fibrillation in cardiosurgical ICU patients

**DOI:** 10.1186/2197-425X-3-S1-A946

**Published:** 2015-10-01

**Authors:** M Arrigo, N Jeger, B Seifert, DR Spahn, D Bettex, A Rudiger

**Affiliations:** Institute of Anesthesiology, University Hospital Zurich, Zurich, Switzerland; Department of Cardiology, University Hospital Zurich / University Heart Center, Zurich, Switzerland; Department of Biostatistics, University Zurich / Epidemiology, Biostatistics and Prevention Institute (ESBI), Zurich, Switzerland

## Introduction

Electrical cardioversion (ECV) of atrial fibrillation (AF) is recommended in patients with hemodynamic instability. ECV may therefore be favorable for critically ill patients with new-onset AF, although evidence is lacking. Data about conversion rates in critically ill patients undergoing urgent ECV are scarce.

## Objectives

The aims of this study were to assess the success of ECV for the treatment of new-onset AF in critically ill patients and to evaluate the stability of sinus rhythm in responders during the subsequent 24 hours.

## Methods

Consecutive cardio-surgical patients with new-onset AF (less than 7 days of duration) treated by ECV were included. All applied shocks were synchronized, of biphasic waveform and performed using an external defibrillator. Physicians were encouraged to administer a high initial energy with escalating doses for subsequent shocks. Repeated shocks within 15 minutes were defined as an ECV session. A conversion into sinus rhythm for at least 30 seconds during an ECV session was defined as a successful ECV. The stability of sinus rhythm during the following 24 hours was investigated and the presence of sinus rhythm at ICU discharge was documented.

## Results

A total of 72 patients were included. Thirty-seven patients had one ECV, the remaining up to 6 sessions during their ICU-stay. Finally, a total of 144 ECV were analyzed in the study. The restoration of sinus rhythm was achieved in 102 (71%) ECV. Hemodynamic instability was present during 117 (81%) ECV. Electrodes were placed in the antero-posterior position for 52% of ECV, 85% of shocks were performed with maximal biphasic energy of 200 Joules. During the 24 hours follow up, the stability of sinus rhythm was poor: after 1 and 24 hours sinus rhythm was documented in only 43% and 23% of patients, respectively. Intravenous amiodarone was administered during the 6 hours before ECV in 94 (65%) cases, but showed no significant effect neither on the immediate success of ECV, nor on the maintenance of sinus rhythm during the first 24 hours. At ICU discharge, 54 (75%) patients were in sinus rhythm, whereby 20% converted spontaneously, 46% after amiodarone post-treatment and only 33% after repeated ECV. The median length of ICU stay was 7 days and ICU mortality of the study population was 15%.

## Conclusions

In this retrospective study, immediate success rate of ECV was 71% and therefore higher than previously reported in critically ill patients. However, early relapse of AF was common, so that only 23% of the patients were still in sinus rhythm after 24 hours. At ICU discharge, 75% of patients were in sinus rhythm, whereby repeated ECV was responsible for a conversion into sinus rhythm in only a third of the patients. Hence, the efficacy of repetitive ECV in restoring sinus rhythm was disappointing.

